# Editorial: Employing Experimental Gnotobiotic Models to Decipher the Host-Microbiota Cross-Talk in Health and Disease

**DOI:** 10.3389/fimmu.2021.729052

**Published:** 2021-08-03

**Authors:** Martin Schwarzer, Helena Tlaskalova-Hogenova, François Leulier, Irma Schabussova

**Affiliations:** ^1^Laboratory of Gnotobiology, Institute of Microbiology, Czech Academy of Sciences, Novy Hradek, Czechia; ^2^Laboratory of Cellular and Molecular Immunology, Czech Academy of Sciences, Prague, Czechia; ^3^Institut de Génomique Fonctionnelle de Lyon, Université de Lyon, Ecole Normale Supérieure de Lyon, Centre National de la Recherche Scientifique, Université Claude Bernard Lyon 1, Unité Mixte de Recherche 5242, Lyon, France; ^4^Institute of Specific Prophylaxis and Tropical Medicine, Medical University of Vienna, Vienna, Austria

**Keywords:** germ-free, host-bacteria interactions, microbiota, gnotobiology, immune system

## Introduction

Eukaryotic organisms have evolved in a world dominated by bacteria and archaea. Rather than face the daunting task of keeping their exposed surfaces germ-free (GF), they have developed close symbiotic relationships. Mucosal surfaces are associated with specific microbial communities that influence various aspects of host physiology and, most importantly, the development and fine-tuning of the immune system. In the last decade, we have witnessed a renewed interest in understanding the role of the microbiota for the homeostasis and disease. This has fostered the development of sophisticated multidisciplinary technologies that enable compositional and functional analysis of the microbiome (1). The most diverse and numerous microbial communities are found in the gastrointestinal tract. Alterations in the gut microbiome and/or disruptions of the cross-talk between host and microbiota has been linked to immune-mediated diseases such as allergies and autoimmune diseases such as inflammatory bowel disease, rheumatoid arthritis, multiple sclerosis and diabetes (2). The aforementioned complexity of the gut microbial ecosystem currently complicates the understanding of the microbiota-host cross-talk, with descriptive reports predominating over mechanistic studies. However, powerful tools for studying host-microbe interactions are germ-free (GF) and gnotobiotic animal models. Although historically mostly rodents and piglets (3) have been used, new vertebrate models, for example fish, and invertabrate models such as *Drosophila* have successfully been developed in recent years (4).

The possibility to colonize GF animals with defined bacterial species or a consortium of bacteria has created a unique opportunity to evaluate the impact of the microbiota on host physiology and immune system (5). Colonization of GF mice with a defined bacterial consortium that mimics the complexity of a specific pathogen-free/specific opportunistic pathogen-free (SPF/SOPF) microbiota has increased the reproducibility of mouse experiments across different institutions and mouse facilities (6). In this regard, Eberl et al. show that one such consortium, Oligo-Mouse-Microbiota12 (Oligo-MM12), could be established in several different animal facilities with excellent reproducibility. Within two weeks after inoculation of the Oligo-MM12 consortium in GF mice similar stable microbial communities were found in all facilities. Furthermore, they showed that a second inoculation of the Oligo-MM12 strains after 72 h was even more effective at establishing a stable microbiota. Thus the reliable *de novo* generation of gnotobiotic rodents contributes greatly to experimental reproducibility, which has significantly benefitted biomedical research in recent years. Using the Oligo-MM12 consortium, Wyss et al. sought to explain the phenomenon behind elevated IgE levels in GF animals. Depending on the mouse facility, mouse model, and breeding conditions, GF mice exhibit elevated total serum IgE levels (6–9). Wyss et al. demonstrated that colonization of mice with a well-defined composition of bacterial species is required to inhibit elevated IgE levels. Features of bacterial consortia that successfully decrease the IgE include presence in early life, acetate production, and immunogenicity reflected in the induction of gut intestinal IgA.

There is a growing interest in the development of microbial-based and microbial-targeted therapies (10). Preclinical studies with “humanized” mice (transfer of human microbiota to germ-free animals) are often necessary methodological requirement to analyze the potential therapeutic effects. This topic was discussed in detail by Rogala et al. GF mice colonized with single wild-type or genetically engineered microbial isolates are an invaluable tool to study the functions of individual bacterial genes and species. GF mice colonized with multiple defined isolates can be used to determine interactions between members of defined consortia. This is elegantly discussed and ways to improve studies of immune-microbial interactions using gnotobiotic mice are presented.

On this theme, Bolsega et al. investigated whether different bacterial minimal consortia affect the outcome of murine norovirus-induced colitis in Il10^-/-^ mice. By comparing two different minimal microbiota (Oligo-MM12 and Altered Schaedler Flora) colonized mice, they show that murine norovirus-triggered colitis depends on the composition of the microbiota. Lengfelder et al. characterized the colitogenic activity of *Enterococcus faecalis* as part of a simplified human microbial consortium based on seven enteric bacterial strains (SIHUMI). They showed that complex bacterial consortium interactions reprogram the gene expression profile and colitogenic activity of the opportunistic pathogen *E. faecalis* towards a protective function. Kostovcikova et al. examined colitis from a nutritional perspective and used a gnotobiotic approach to show that diet rich in animal protein exacerbates acute dextran sulfate sodium (DSS)-induced colitis, whereas diet rich in plant protein does not. They concluded that interactions between a dietary protein of animal origin and the gut microbiota increase sensitivity to intestinal inflammation by promoting the pro-inflammatory responses of monocytes.

The gut microbiota can influence brain functions and behavior, including hypothalamic-pituitary-adrenocortical axis (HPA) activity. Using SPF and GF male BALB/c mice, Vagnerova et al. investigated the influence of the microbiota on the acute restraint stress (ARS) response in the pituitary, adrenal gland, and intestine, an organ of extra-adrenal glucocorticoid synthesis. They showed that GF animals have an exaggerated HPA response to stress.

Finally, Murdoch and Rawls review the key insights provided by the gnotobiotic zebrafish model on the effects of microbiota on innate immunity. This included evidence that the perception of and response to the microbiota is evolutionarily conserved. They describe how to strengthen the zebrafish model system and provide new insights into the host-microbe interactions that would be difficult to study in mammalian models.

Overall, we have assembled a set of eight research articles that bring new insights into host-microbiota interactions. These studies all highlight the advantages of gnotobiotic rodent and fish models, and demonstrate the modularity of defined bacterial minimal consortia that can be used to achieve greater standardization of biomedical research across different animal facilities.

## Author Contributions

All authors listed have made a substantial, direct and intellectual contribution to the work, and approved it for publication.

## Funding

This work was supported by grant 18–07015Y from the Czech Science Foundation and EMBO Installation grant to MS, The Danube-ARC funded by the Government of Lower Austria to IS and Czech Health Research Council grant NV19-03-00179 to HT-H.

## Conflict of Interest

The authors declare that the research was conducted in the absence of any commercial or financial relationships that could be construed as a potential conflict of interest.

## Publisher’s Note

All claims expressed in this article are solely those of the authors and do not necessarily represent those of their affiliated organizations, or those of the publisher, the editors and the reviewers. Any product that may be evaluated in this article, or claim that may be made by its manufacturer, is not guaranteed or endorsed by the publisher.
